# Editorial: Evolving Prospects of Bovine Respiratory Diseases and Management in Feedlot Cattle

**DOI:** 10.3389/fvets.2022.854844

**Published:** 2022-02-25

**Authors:** Annamaria Pratelli, Barbara Padalino

**Affiliations:** ^1^Department of Veterinary Medicine, University of Bari Aldo Moro, Bari, Italy; ^2^Division of Animal Sciences, Department of Agricultural and Food Sciences, Alma Master Studiorum, University of Bologna, Bologna, Italy

**Keywords:** bovine, respiratory diseases, health, management, prevention, welfare

Bovine respiratory disease (BRD) is one of the major economical and welfare concerns for the cattle industry worldwide (1). BRD incidence varies enormously according to farm management, prophylaxis measures, involved pathogens, and animal-related predisposing factors since all of them play a decisive role in the development and severity of the disease (2, 3). The Research Topic consequently aims to broaden the current knowledge on BRD etiology, on its pathogenetic mechanisms, on possible hazards related to breeding and transport practices, on immunopathological implications, on new technologies for the diagnosis, on possible prophylaxis and treatment, as well as on risk factors and on emerging antimicrobial resistance (AMR). Due to the multifactorial etiology of the syndrome, the articles encompass within this Research Topic have all a multidisciplinary approach.

Among microorganisms involved in BRD, *Pasteurella multocida* plays a primary role. An interesting study by Zhan et al. elucidated the toxicity targets of *P. multocida* serogroup A providing fundamental information on the pathogenic mechanism and on the antimicrobial drugs resistance of this pathogen of BRD. Similarly, *Mannheimia haemolytica* is involved in BRD onset. Severe infections are often characterized by dysregulated inflammatory responses in the lungs, where IL-17A plays a key role in the inflammatory response activating innate and adaptive immune cells and exacerbating lung congestion. Two independent studies carried out by Slate et al. supported the hypothesis that IL-17A signaling may contribute to lung immunopathology and that further understanding of this inflammatory pathway during BRD could expand therapeutic intervention strategies for managing BRD.

Notoriously, *bovine coronavirus* (BCoV) is one of the most common agents involved in BRD. In the study reported by Kiser and Neibergs several positional candidate genes were identified in association with BRD and BCoV in dairy calves and feedlot cattle in the USA. This study allows to better elucidate the etiology of the disease and allows the identification of loci to be considered for genomic selection, suggesting that selection may reduce susceptibility to BCoV infection and BRD. Oliveira et al. also demonstrated that in addition to the common pathogens, other microorganisms may contribute to the onset of the syndrome. Immunohistochemical assays on formalin-fixed paraffin-embedded pulmonary sections have identified, among the other, *malignant catarrhal fever virus* (MCFV), as well as *Mycoplasma bovis*, in single or mixed infections, as the most frequent pathogen, expanding the list of primary agents in the development of BRD. Moreover, a retrospective study on fatal calf pneumonia in Italy during 2009–2019, allows the identification of *M. bovis* either as the single agent or as a concurrent agent with pathogens correlated with BRD, turning the spotlight on *M. bovis* infection and on control measures to apply for the prevention of lethal pneumonia outbreaks in dairy herds (Fanelli et al.).

A 1-year cross-sectional study by Padalino et al. on 169 beef steers documented the prevalence of the aforementioned BRD-related pathogens and the manifested clinical signs before and after a long journey from France to Italy, identifying contextually the possible predisposition factors. The survey demonstrated that the animals displaying clinical signs and positive for the most common pathogens involved in BRD increased dramatically at arrival, that the transport favored coinfections and that the weather conditions were predisposing factors for many of the pathogens. The study also demonstrated that in the majority of the cases co-infections were present and highlighted that understanding of factors responsible for increasing the likelihood of BRD can be useful to reduce or minimize the incidence of the syndrome and to implement animal transport regulations.

Due to its multifactorial etiology, the diagnosis of BRD is still challenging. Since early diagnosis is crucial, in this special issue new diagnostic tools have been presented. A specific one-step multiplex real-time PCR assay was developed for simultaneous detection of five respiratory disease viruses involved in BRD without cross-reaction with others. The test proved to have good specificity and sensitivity allowing rapid detection of pathogens to guide the formulation of BRD prevention and control measures (Zhang et al.). Lastly, in addition to the current laboratory methods for detecting BRD infected calves, thoracic ultrasonography (TUS) was investigated in clinical settings by Porter et al. TUS proved to be able to identify calves with abnormal lung pathology that would have otherwise been misclassified and can provide additional information on calf health due to the high correlation with lung pathology at necropsy. TUS could be consequently recommended on arrival after long-distance journeys to give an early diagnosis and allow timely treatment.

Prevalence and risk factors for the main bacterial pathogens affecting the respiratory tract of calves from the spring processing to the reprocessing at feedlots were also investigated by Nobrega et al. identifying in *P. multocida* the most prevalent species, regardless of time point, but with an increase in prevalence at the weaning/induction sampling. Comingling and co-location of feedlots were not associated with the prevalence of any respiratory pathogen. Contextually, the AMR profile of the four species was characterized phenotypically and genotypically, and limited evidence support increased resistance to respiratory bacteria from the spring processing to reprocessing at feedlots with a few exceptions. On the contrary, parenteral use of macrolides as metaphylaxis at the feedlot induction was associated with an increased minimum inhibitory concentration (MIC) against macrolides in *P. multocida, M. haemolytica*, and *Histophilus somni* contributing to historical changes in macrolides MIC data of respiratory bacteria of beef cattle. Overall, the AMR phenotypes were corroborated by the presence of AMR genes. Prevalence and epidemiology of AMR was also investigated by Andrés-Lasheras et al. on beef cattle upon arrival at Canadian feedlot and before antimicrobials were administered. *M. haemolytica, P. multocida*, and *H. somni* with multidrug-resistant (MDR) profiles were more often isolated from dairy-type than from beef-type cattle, showing that the latter presented higher odds of AMR bacteria as compared to auction-derived calves and that resistance to oxytetracycline was most frequently observed across all *Pasteurellaceae* species and cattle types.

Despite the increasing use of prophylaxis measures, feedlot placement remains a high-risk period for calves to develop respiratory disorders, and new therapy should be proposed. Interestingly, Tan et al. demonstrated that ginsenoside Rb2 and Rb3, major pharmacological ingredients in the plants of ginseng, were able to inhibit the replication and proliferation of bovine and swine pestiviruses, suggesting their potential for effective treatment against infection and thus representing a possible alternative to the use of antibiotics.

These experimental prospective and retrospective studies and critical reviews speculate that the role of the viral infections as the starting point for BRD on which secondary opportunistic bacteria enter, was only a simplistic view of the pathogenesis of the disease, while a primary role of some pathogens rarely detected in the past and generally considered of minor importance, as well as several cofactors such as management of transport and breeding, were identified in eliciting the disease. The potential pathogenetic role for these pathogens and the high frequency with which co-infections occur, make BRD a complex disease difficult to control (4). Despite the increasing use of prophylaxis and treatment measures, feedlot placement remains a high-risk period for calves to develop respiratory disorders, also considering that viral shedding is often greatest even before animals become symptomatic. Consequently, the most important aspects to counteract infections and to restrain the development of BRD and spreading of AMR remain the proper management of cattle, the development of new technologies for early diagnosis, and the application of appropriate prophylaxis measures, since minimizing the risk and the incidence of BRD is crucial to improve cattle productivity, health, and welfare (3, 5, 6). We are proud and highly motivated to promote the development of evidence-based guidelines to prevent BRD in the Frontiers open source venue, and future article collections are needed to further address the gaps of knowledge highlighted in this special issue.

## Author Contributions

All authors listed have made a substantial, direct, and intellectual contribution to the work and approved it for publication.

## Conflict of Interest

The authors declare that the research was conducted in the absence of any commercial or financial relationships that could be construed as a potential conflict of interest.

## Publisher's Note

All claims expressed in this article are solely those of the authors and do not necessarily represent those of their affiliated organizations, or those of the publisher, the editors and the reviewers. Any product that may be evaluated in this article, or claim that may be made by its manufacturer, is not guaranteed or endorsed by the publisher.
